# Dietary Inclusion of Casimiroa (*Casimiroa edulis* L) Seed Kernel Meal on Meat Qualities, Blood Profiles, and Cecal Microbiota of Broiler Chicken

**DOI:** 10.1155/vmi/2733198

**Published:** 2026-08-13

**Authors:** Mohammedjuhar Musa Adame, Mengistu Urge Letta, Biazen Abrar Bedru, Sileshi Gadissa Terfa, Meseret Girma Abebe, Misba Alewi Abdu

**Affiliations:** ^1^ School of Animal and Range Sciences, Haramaya University, Dire Dawa, Oromia, Ethiopia, haramaya.edu.et; ^2^ Debre Zeit Agricultural Research Centre, Ethiopian Institute of Agricultural Research, Bishoftu, Oromia, Ethiopia, eiar.gov

**Keywords:** broilers, Casimiroa seed kernels, cecal microbiota, meat quality

## Abstract

The purpose of this study was to evaluate the effects of dietary inclusion of soaked Casimiroa seed kernel (CSK) meal on broiler meat quality, blood parameters, immunological response, and cecal microbiota. A total of 192 Cobb‐500 broilers were assigned to four dietary treatments (0%, 1.5%, 3.0%, and 4.5% soaked CSK) in a completely randomized design with four replicates and 12 birds per replicate. The birds were fed isocaloric and isonitrogenous diets for 42 days. Majority of the optimal results were observed at the 1.5% and 3.0% soaked CSK inclusion levels, as evidenced by significant quadratic trends. Breast and thigh meat quality showed quadratic improvements in protein content, fat reduction, drip and cooking loss reduction, redness, and sensory scores (juiciness and tenderness). Key blood parameters (red blood cells, hemoglobin, total protein, immunoglobulins, and globulins) were improved quadratically, while serum triglycerides and cholesterol decreased linearly. Additionally, all soaked CSK inclusion levels quadratically reduced pathogenic E. coli and coliforms without impacting beneficial Lactobacilli and Enterococcus spp. Based on these results, dietary inclusion of soaked CSK meal up to 3.0% is a safe and effective strategy for simultaneously enhancing broiler meat physicochemical quality, improving metabolic and immune status, and promoting a beneficial gut microbiota balance.

## 1. Introduction

Poultry industry has become a dominant global source of animal meat protein due to its affordability, nutritional benefits, and widespread cultural and religious acceptance [1]. This rising demand drives continuous improvements in feeding regimens and alternative feed ingredients to optimize growth, enhance meat quality, and support chicken health. Following the European Union ban on antibiotic growth promoters (AGPs) in 2006 [2], interest in natural alternatives with diverse biological functions has increased. This has prompted a critical search for economically viable, locally available, and nutritionally adequate feed ingredients [3]. The shift away from AGPs is further justified by public health concerns regarding residue accumulation in meat, meat products, and the environment. Concurrently, growing consumer demand for safe, superior quality, and affordable meat has directed attention toward natural alternatives, such as spices, herbs, and plant extracts derived from leaves, roots, fruits, and seeds, in poultry feeding, thus ensuring product acceptability [4].

The incorporation of phytogenic fruit seed derivatives of different herbs as alternative feed supplements has gained significant scientific interest in poultry nutrition due to their multibioactive properties, cost‐effectiveness, and favorable safety profile [5, 6]. These underutilized byproducts offer a sustainable strategy to enhance broiler chicken physiological performances, improve product quality, and support gastrointestinal health [5]. Their application is considered safe and economically effective, as they are readily available, edible, and rich in nutrients, thereby benefiting both birds and consumer health [6]. Furthermore, specific studies have demonstrated that certain fruit seeds can bolster endogenous antioxidant systems, improve nutrient utilization, and enhance meat quality by modulating lipid metabolism [7, 8].

Before incorporation into commercial diets, alternative feed ingredients require evaluation beyond basic nutrient analysis to a holistic assessment of their implications for broiler health, product quality, and overall food safety [9]. Many such ingredients encompass antinutritional factors, which may impair nutrient utilization, growth performance, blood biochemistry, and organ function, while also degrading meat quality [9, 10]. Additionally, the gut microbiota, a critical mediator of host metabolism and health, is also highly sensitive to dietary changes [11]. Therefore, a holistic assessment encompassing physiological, product quality, and microbiological parameters is essential for the evaluation of any new feed ingredient.

Ethiopia possesses extensive land suitable for cultivating Casimiroa edulis L. This drought‐tolerant species grows across an altitudinal range of 600–2700 m, contributing to its widespread availability and high productivity in the country [12, 13]. The tree is commonly integrated into home gardens and agroforestry systems, where it provides livestock feed, fruit, fuel, and shade for coffee [14, 15]. Additionally, its use as an ornamental shade tree in urban and periurban areas further supports large‐scale fruit and seed production [16]. A mature Casimiroa tree can yield approximately one ton of fruit annually [17, 18]. Seeds constitute about 23.68% of the total fruit mass, indicating a considerable yearly supply of seed kernels as a byproduct [18]. Although the fruit pulp is edible, large quantities of the kernels are discarded, especially in juice processing facilities [12]. Given the kernel’s abundance and current underutilization as a byproduct in Ethiopia, exploring its incorporation into poultry feed presents an economically attractive opportunity, positioning it as a feasible yet overlooked feed ingredient.

Casimiroa edulis L. (family Rutaceae), traditionally recognized for its medicinal properties, represents a potential phytogenic feed additive for use in poultry nutrition. Preliminary proximate analysis indicates that processed Casimiroa seed kernel (CSK) meal contains a metabolizable energy (ME) value of 3571.26 kcal/kg DM and a crude protein content of 12.48%, supporting its potential nutritional utility [19]. However, raw kernels harbor antinutritional factors, specifically water‐soluble alkaloids, tannins, and oxalates, that can impair nutrient utilization and raise safety concerns [20, 21]. Among various processing methods, soaking has proven more effective than boiling: it reduces tannins by 84.31%, oxalates by 37.08%, and total alkaloids by 47.43%, whereas boiling achieves only a 32.87% reduction in total alkaloids [22]. Such treatment enhances the feeding suitability of CSK meal. Even though with these antinutritional challenges, Casimiroa species are recognized for their unique bioactive compounds, including high levels of unsaturated fatty acids (94.2%), phenolic compounds, flavonoids, alkaloids, coumarins, and zapotin, all of which exhibit documented pharmacological properties [17, 20, 21, 23]. Numerous studies have reported that these compounds confer a broad spectrum of biological activities, such as antioxidant, antimicrobial, anti‐inflammatory, immunomodulatory, and hepatoprotective effects [20, 21, 24].

Previous studies in rats, dogs, and guinea pigs have demonstrated that Casimiroa edulis seed extracts exert significant cardiovascular effects, primarily through blood pressure reduction and vasorelaxation [25–28]. Pharmacological investigations using aqueous and alcoholic extracts of the seeds have further reported improvements in blood pressure, cardiac activity, respiratory rate, muscular tone, and electroencephalographic activity in various animal models, including dogs, cats, rabbits, and guinea pigs [29]. More recently, Corona et al. [24] found that supplementing laying hens with 5 g/kg of nanoencapsulated Casimiroa edulis fruit meal for 1 month caused no cellular toxicity while enhancing cell proliferation, immune response, and antioxidant activity.

Despite the recognized medicinal properties of CSK meal, limited information exists regarding its nutritional effects on meat quality attributes, blood biochemistry, immune function, and cecal microbial communities in broilers, warranting further investigation into its potential influence on broiler physiology and meat quality traits. Therefore, we hypothesize that increasing dietary inclusion of processed CSK meal (0%–4.5%) will induce significant linear, quadratic, or cubic trends in meat quality or physiological parameters, indicating that the meal can be incorporated up to the tested levels to determine dose‐dependent effects. Accordingly, this study aimed to evaluate the dose‐dependent effects of dietary inclusion of processed CSK meal on meat quality, hematology, serum biochemistry, immune function, and cecal microbial communities in broiler chickens.

## 2. Materials and Methods

### 2.1. Study Area

This experiment was carried out at Haramaya University’s poultry farm, which is located 515 km east of Addis Ababa, Ethiopia. The experimental site lies at an elevation of 2006 m above sea level, with geographic coordinates of 9°41′N latitude and 42°4′E longitude. The area receives an average annual rainfall of 790 mm and has an average temperature of 17°C, with minimum and maximum temperatures of 14°C and 23.4°C, respectively [30].

### 2.2. Source and Processing of CSKs

Casimiroa fruit seeds were used as a novel feed ingredient. The fruits were obtained from commercial vendors at Dengego Market in Maya City, Eastern Hararghe, Ethiopia. These vendors sourced their produce from local cultivators across multiple localities within the area. Seed kernels were collected from the fruits. To reduce the crude fiber content, the seed coats were manually removed. The isolated kernels were rinsed with tap water to eliminate surface contaminants. According to Elkady et al. [23], whole Casimiroa seed meal contains 53.73 mg/g of tannins. To lower antinutritional factors, the kernels were manually chopped and soaked in cold water for 24 h [31]. After soaking, the chopped seeds were sun‐dried for 4 days (duration dependent on solar intensity). The dried seeds were then ground to a 5‐mm particle size using a grinding machine, yielding the final processed CSK meal. The product was stored in an airtight container to maintain quality and prevent spoilage.

### 2.3. Proximate Analysis of Primary Feed Ingredients

Before formulating the experimental diets, representative samples of all primary ingredients such as corn, soybean meal, soaked CSK meal, Noug seed cake, and wheat shorts were subjected to chemical analysis at the Animal Nutrition Laboratory of Haramaya University. A standardized proximate analysis was conducted to determine the content of dry matter, crude fiber, ash, ether extract, calcium, and phosphorus in each ingredient (Table 1). Nitrogen content was quantified using the Kjeldahl method [32], and crude protein was derived as *N* × 6.25. Calcium and phosphorus concentrations were measured by atomic absorption spectroscopy and spectrophotometry, respectively [32]. The ME of the diets was calculated according to the equation established by Wiseman [33]: ME (kcal/kg DM) = 3951 + 54.4 EE − 88.7 CF − 40.8 Ash.

**TABLE 1:** Proximate analysis of primary feed ingredients (based on DM%).

Ingredients	DM%	CP	CF	EE	Ash	Ca	P	ME (Kcal/kg DM)
Maize	90.32	9.45	4.16	3.76	4.67	0.07	0.46	3595.72
Wheat shorts	90.94	15.35	9.67	3.48	4.74	0.16	0.60	3089.19
Soybean meal	93.31	39.44	8.62	4.21	5.97	0.34	0.56	3171.72
Noug seed cake	92.06	30.45	16.48	7.41	10.8	0.33	0.58	2451.46
CSK meal	89.08	12.48	2.94	0.96	4.19	1.01	0.11	3571.26

*Note:* kcal = kilocalorie. P = phosphorus, Ca = calcium.

Abbreviations: CF = crude fiber, CP = crude protein, CSK = Casimiroa seed kernel, DM = dry matter, EE = ether extract, ME = metabolizable energy.

### 2.4. Experimental Ration Formulation and Design

Based on the laboratory chemical analysis results, four iso‐nitrogenous and isoenergetic dietary treatments were formulated with WinFeed 2.8 software to meet established broiler nutritional requirements [34]. Separate starter and finisher diets were prepared (Table 2) and processed at the Haramaya Area Union Animal Feed Processing Centre. Representative samples from each treatment were subsequently analyzed to verify that the nutritional specifications were satisfied. The study followed a completely randomized design (CRD) with four dietary treatments. These treatments were formulated by substituting soaked CSK meal for a portion of the primary energy ingredients (maize and wheat shorts) in the concentrate mixture, based on its comparable energy value. A total of 192 unsexed Cobb‐500 broiler chicks were randomly allocated to the four treatments, with four replicates per treatment and 12 birds per replicate. Inclusion rates for processed CSK meal were set at 0% (T1, control), 1.5% (T2), 3% (T3), and 4.5% (T4) of the concentrate. These levels were conservatively determined due to the absence of prior inclusion standards for soaked CSK meal in broiler nutrition and its reported moderate fiber and phytochemical content [21]. Consequently, the maximum inclusion was capped at 4.5%, a level within the low‐to‐moderate range recommended for novel fruit byproducts in poultry feeds to mitigate potential antinutritional effects [35].

**TABLE 2:** Proportion of ingredients (%) and proximate analysis of starter and finisher broiler rations.

Ingredients	Starter				Finisher			
	T1	T2	T3	T4	T1	T2	T3	T4
Maize grain	47	46.5	46.5	45	58	57.5	56	54.5
Soybean meal	30.25	30.25	30.25	30.25	31.5	31.5	31.5	31.5
Noug seed cake	16.25	16.25	16.25	16.25	4.8	4.8	4.8	4.8
Wheat shorts	3.5	2.5	1	1	2.5	1.5	1.5	1.5
CSK meal	0	1.5	3	4.5	0	1.5	3	4.5
Limestone	1.5	1.5	1.5	1.5	1.5	1.5	1.5	1.5
Vitamin premix	0.5	0.5	0.5	0.5	0.5	0.5	0.5	0.5
Lysine	0.15	0.15	0.15	0.15	0.15	0.15	0.15	0.15
Methionine	0.25	0.25	0.25	0.25	0.25	0.25	0.25	0.25
DCP	0.3	0.3	0.3	0.3	0.3	0.3	0.3	0.3
Salt	0.3	0.3	0.3	0.3	0.5	0.5	0.5	0.5
**Total**	**100**	**100**	**100**	**100**	**100**	**100**	**100**	**100**

*Proximate analysis (%)*								
Dry matter	89.58	89.83	89.15	88.77	87.64	88.74	88.31	88.54
Crude protein	21.87	22.03	21.91	21.96	19.67	19.74	19.92	20.01
Crude fiber	8.43	8.18	7.99	7.95	8.03	7.98	7.99	8.05
Ether extract	4.89	4.75	4.57	4.39	4.38	4.28	4.53	4.59
Ash	7.57	8.15	8.12	8.31	6.6	6.54	6.91	7.02
Calcium	0.39	0.62	0.68	0.65	0.47	0.34	0.73	0.81
Phosphorus	0.45	0.48	0.51	0.37	0.31	0.38	0.31	0.42
ME (Kcal/kg DM)	3160.89	3150.67	3159.62	3145.13	3207.96	3209.3	3206.81	3200.38

*Note: T*1 = 0% soaked CSK meal, *T*2 = 1.5% soaked CSK meal, *T*3 = 3% soaked CSK meal, *T*4 = 4.5% soaked CSK meal. The bold values denote the column sums, indicating that all ingredient proportions within each respective column total 100%.

Abbreviations: CSK = Casimiroa seed kernel, DCP = Dicalcium phosphate.

### 2.5. Experimental Bird Management

Upon arrival, chicks received a sugar solution in drinking water to alleviate transport stress. Each replicate group was housed in a deep‐litter pen (1.75 × 1 m) containing teff straw litter at a 7‐cm depth. All equipment was cleaned and sterilized with formalin prior to the experiment. Ambient temperature was maintained at 32°C for the first week and subsequently reduced by 2°C weekly until reaching 22°C, regulated by a dimmer switch controlling a 250‐W infrared bulb and monitored with a digital hygrometer. Feed was offered ad libitum twice daily, with continuous access to clean water. Body weight was measured at the start, weekly during the trial, and at the end using a precision scale. Strict biosecurity measures were maintained throughout the study. The chicks received an HB1 Newcastle disease vaccine on day 7, followed by a Lasota booster via eye drops on day 21. Additionally, they were vaccinated against infectious bursal (Gumboro) disease as per the manufacturer’s guidelines. Throughout the experiment, uniform health and biosecurity measures, along with consistent management practices for lighting, brooding, feeding, and watering, were implemented across all treatment groups, except for the dietary variations.

### 2.6. Data Collection

#### 2.6.1. Meat Sample Preparation

At the end of the feeding trial, four birds were randomly selected from each replicate. The selected birds were fasted for 12 h to clear the digestive tract and then weighed. Subsequently, each bird was restrained using a killing cone and humanely exsanguinated by severing the neck, ensuring rapid and complete bleeding. Following the slaughtering process, a total of 64 meat samples (breast and thigh) were collected from each bird to assess physical quality, nutritional composition, and sensory attributes. The breast and thigh samples were placed in zipper‐sealed plastic bags and stored in a cold room at 4°C for 12 h prior to analysis. These samples were used to determine meat physical quality parameters (color, cooking loss, drip loss), nutritional composition (dry matter, protein, fat, ash), and sensory evaluation conducted by a consumer panel.

#### 2.6.2. Meat Physical Quality Evaluation

For instrumental meat color measurement, a portable Hunter laboratory colorimeter (Mini Scan EZ spectrophotometer) was used to measure the three numerical color measurements lightness (*L* ^∗^), redness (*a* ^∗^), and yellowness (*b* ^∗^), respectively [36]. The spectrophotometer was calibrated before analyzing the samples against white and black standards. Measurements were reported as *L* ^∗^, *a* ^∗^, and *b* ^∗^ values based on CIE standards [36]. Color evaluations comprised the average of three measurements taken from the upper, middle, and lower regions of the breast and thigh muscles, ensuring that samples were free from defects such as blood vessels that could distort color readings.

On the other hand, meat water holding capacity was indirectly measured from drip and cooking losses. Drip loss was determined using the suspension method of [37]. About 40‐g meat samples taken from each breast and thigh were suspended in a plastic bag, ensuring that there was no contact between the sample and the bag. Samples were kept in the cold room at 4°C for 24 h. The weight of each sample was recorded before and after being suspended. Drip loss was expressed as a percentage of weight loss after suspension relative to the initial weight of the slice. Cooking loss was measured on a 40‐g sample from each breast and thigh muscles; then, each sample was put in a zipper plastic bag and cooked in the thermostatically controlled water bath set at 80°C for 60 min as described by Hoffman et al. [38]. Then, meat samples were cooled by running tap water and samples were taken from the bags, dried with cotton sheet, and reweighed. Both drip and cooking losses were calculated using the formula:

(1) loss %=initial weight−final weightinitial weight∗100.

#### 2.6.3. Meat Nutritional Composition Analysis

The breast and thigh meat samples used for color evaluation were subsequently used for proximate composition analysis according to the methods of AOAC [26]. The samples were minced, wrapped in aluminum foil, and partially dried at 55°C for 72 h. Partially dried meat samples were ground, and the weighed sample was dried in an oven at 105°C for 12 h to determine dry matter. Crude protein was derived from the nitrogen content, quantified via the Kjeldahl method, using a conversion factor of 6.25. The ash content was determined by incineration in a muffle furnace at 550°C for 6 h.

#### 2.6.4. Consumer Panellist’s Sensory Evaluation

Sensory attributes’ evaluation was conducted by consumer panellists. A total of 16 consumer panellists were selected from experienced staff working in food science, meat and dairy science, and technology laboratories of Haramaya Universities and postgraduate students. Panellists were selected based on their meat consumption habits or experiences, sensory capability, health conditions, and free from any addictions. Accordingly, sensory attributes including tenderness, juiciness, flavor, and overall acceptability were evaluated on 64 meat samples. Prior to roasting, the meat samples were thawed and cut into 2.5‐cm cubes. Then, the samples were wrapped in aluminum foil and cooked in an oven at 175°C for 45 min. After roasting, the meat samples were cooled at room temperature to a level of warm state and set directly on the plates and then offered to panellists in a sequential monadic manner. Each panellist involved in the taste was given a score sheet, where they marked their findings of the sensory attributes of the meat samples. Warm water was provided to the panelists; the palates were cleansed between samples and paused for 30 s. Each panelist tested four samples and scored them according to Damaziak [39], using 7‐point hedonic scales. The attributes assessed were tenderness (7 = very tender to 1 = very though), juiciness (7 = very juicy to 1 = very dry), chicken aroma and flavor intensity (7 = very desirable to 1 = very undesirable), and overall liking (7 = like very much to 1 = dislike very much).

#### 2.6.5. Blood Collection and Analytical Methods

On day 42 of the experiment, 5‐mL blood samples were collected from the wing veins of four broiler chickens per replicate, specifically chosen for meat quality evaluation. Each sample was subdivided: 2.5 mL was transferred to a tube containing ethylene diamine tetra‐acetic acid (EDTA) as an anticoagulant for hematological analysis, and 2.5 mL was placed in a tube without anticoagulant for serum preparation. In the EDTA‐treated blood, total red blood cell (RBC) and white blood cell (WBC) counts were performed using a hemocytometer [40]. Hemoglobin (Hb) concentration was determined by the actin hematin method, and packed cell volume (PCV) was assessed via the microhematocrit technique. The mean corpuscular volume (MCV), mean corpuscular hemoglobin (MCH), and mean corpuscular hemoglobin concentration (MCHC) were calculated as described [41]. The plain tubes were centrifuged at 3000 rpm for 15 min to obtain serum, which was stored at −20°C. Serum alanine aminotransferase (ALT), aspartate aminotransferase (AST), and alkaline phosphatase (ALP) activities were measured according to established methods [42]. Concentrations of cholesterol, glucose, and triglycerides were determined using standard enzymatic procedures [43]. Total serum protein was quantified using a refractometer [31]. Total immunoglobulin was assessed via the zinc sulfate turbidity test Mondesire [44], wherein the optical density of test and control samples was measured at 545 nm with a spectrophotometer.

#### 2.6.6. Determination of Cecal Bacterial Load

On day 42 of the growth experiment, cecal samples were taken from 64 birds that underwent meat quality evaluation. The sampled birds comprised 16 from each treatment and four from each replicate. They were selected randomly and slaughtered procedurally for bacterial load determination [45]. Following identification and ligation, the ceca were gently squeezed to aseptically transfer their contents into a sterile plastic bag for transport to the laboratory on ice. For analysis, 10 g of content from each treatment replicate was weighed in duplicate into a sterile container. Each sample was diluted in 90 mL of 1% phosphate‐buffered saline (PBS), homogenized thoroughly using a vortex mixer, and serially diluted to a final range of 10^−2^–10^−6^. One milliliter of each serially diluted sample was transferred into a respective labeled Petri dish. Enumeration of Enterococcus spp., Lactobacillus, E. coli, and total coliforms was performed using Slanetz and Bartley Agar, MRS Agar (deMan, Rogosa and Sharpe), MacConkey Agar (MA), and Violet Red Bile Glucose Agar (VRBGA), respectively [46, 47]. The specific media was prepared according to the manufacturer’s instructions and poured into Petri dish containing serial diluted sample and allowed to solidify. The solidified culture plates were incubated at 37°C for 24 h for Enterococcus spp., E. coli, and coliforms aerobically [46] and at 37°C for 48 h anaerobically for lactobacillus [48]. The dilution plate showed well bacterial growth with 30–300 colonies selected, counted using automated colony counter to determine colony forming unit per gram (cfu/g) of the cecal samples for each duplicate, and then converted to log10 for statistical analysis as per the method described by Oyeagu et al. [49].

### 2.7. Statistical Analysis

Data were analyzed using JMP Pro version 17.2 (SAS Institute Inc., Cary, NC, USA). A one‐way analysis of variance (ANOVA) was performed using the general linear model (GLM) procedure. When significant treatment effects were detected (*α* = 0.05), means were separated using Tukey’s honest significant difference (HSD) test. Orthogonal polynomial contrasts were applied to evaluate dose‐dependent responses to increasing levels of dietary CSK meal, testing for linear, quadratic, and cubic trends. All results are reported as treatment means ± standard error of the mean (SEM), accompanied by *p*‐values from both ANOVA and orthogonal contrast tests. The statistical model used was *Y*_*i* *j*_ = *μ* + *T*_*i*_ + *ε*_*i* *j*_, where *Y*_*i* *j*_ represents the observed value for the *j*th experimental unit nested within the *i*th treatment, *μ* is the overall mean, Ti is the fixed effect of the *i*th CSK meal inclusion level (*i* = 0, 1.5, 3.0, or 4.5%), and *ε*_*i* *j*_ denotes the residual error.

## 3. Results

### 3.1. Meat Physicochemical Quality Parameters

The inclusion of soaked CSK meal into broiler diets significantly influenced selected meat physicochemical quality parameters (Table 3). For physical meat quality, instrumental redness (*a* ^∗^ value) was significantly altered (*p* < 0.05) in both breast and thigh muscles. Breast meat redness followed a significant quadratic trend, with higher values at T2 and T3. Thigh meat redness exhibited linear, peaking at T3. Lightness (L) and yellowness (b) were unaffected by treatment (*p* > 0.05). Water‐holding capacity showed a strong quadratic relationship with CSK inclusion, with lower and intermediate levels (T2, T3) yielding the lowest cooking and drip losses. The control (T1) and highest inclusion level (T4) resulted in the greatest losses. Regarding nutritional composition, dry matter and ash content were unaffected (*p* > 0.05). However, CSK supplementation significantly altered key macronutrient profiles following polynomial trends. Crude protein in breast meat increased quadratically in T2 and T3. Similarly, the ether extract content in both muscles decreased quadratically at intermediate CSK levels.

**TABLE 3:** Effects of dietary inclusion of soaked CSK meal on the physicochemical quality parameters of breast and thigh meat of Cobb‐500 broiler chicks.

Parameters		Treatments				SEM	*p* value			
		T1	T2	T3	T4		ANOVA	Linear	Quadratic	Cubic
*Physical meat quality analysis*										
Breast Color	*L* ^∗^	50.64	53.15	50.08	54.78	1.55	0.35	0.31	0.30	0.28
	*a* ^∗^	3.96^b^	4.86^a^	4.80^a^	2.61^b^	0.54	0.04	0.18	0.01	0.64
	*b* ^∗^	12.23	12.13	11.44	10.12	0.58	0.09	0.48	0.30	0.99

Thigh Color	*L* ^∗^	49.29	50.08	49.99	48.87	1.95	0.97	0.87	0.62	0.99
	*a* ^∗^	4.14^b^	4.87^ab^	5.85^a^	4.37^b^	0.49	0.02	0.02	0.05	0.37
	*b* ^∗^	9.08	9.63	8.81	9.68	0.76	0.81	0.77	0.84	0.38

Breast WHC	Coking Loss (%)	30.38^a^	27.25^b^	27.45^b^	31.37^a^	1.06	0.04	0.61	0.004	0.94
	Drip Loss (%)	2.31^a^	1.19^b^	1.23^b^	2.83^a^	0.44	0.04	0.70	0.004	0.92

Thigh WHC	Coking Loss (%)	31.95^a^	29.34^b^	29.49^b^	32.73^a^	0.87	0.04	0.63	0.004	0.93
	Drip Loss (%)	2.03^a^	1.59 ^ab^	1.49^b^	2.32^a^	0.19	0.03	0.49	0.005	0.51

*Meat Nutritional Composition Analysis*										
Breast composition	Dry matter	26.25	26.62	26.62	26.05	0.25	0.43	0.11	0.78	0.61
	Crude protein	22.00^b^	23.18^a^	23.85^a^	21.75^b^	0.39	0.02	0.11	0.005	0.88
	Ether extract	2.48^a^	1.96^b^	1.74^b^	2.5^a^	0.16	0.009	0.22	*p* < 0.001	0.77
	Ash	1.24	1.27	1.26	1.26	0.02	0.55	0.49	0.35	0.39

Thigh composition	Dry matter	27.88	27.86	27.86	27.07	0.16	0.75	0.41	0.47	0.78
	Crude protein	18.17	19.50	19.47	17.81	0.47	0.05	0.71	0.02	0.11
	Ether extract	7.41^a^	7.01^ab^	6.43^b^	7.52^a^	0.16	*p* < 0.001	0.36	*p* < 0.001	0.37
	Ash	1.04	1.07	1.06	1.06	0.02	0.55	0.49	0.35	0.39

*Note:* a, b = means within a row bearing different superscript are significantly different (*p* < 0.05). *L* ^∗^ = lightness, *a* ^∗^ = redness, *b* ^∗^ = yellowness, T1, T2, T3, and *T*4 = 0, 1.5, 3, and 4.5 level comprise Casimiroa seed kernel meal rations, respectively.

Abbreviations: SEM = standard error of the mean, WHC = water holding capacity.

### 3.2. Sensory Evaluations of Breast and Thigh Meat

Sensory analysis indicated that the inclusion of soaked CSK meal in broiler diets did not alter the aroma, flavor, or overall acceptability of breast and thigh meat (*p* > 0.05). Significant effects were confined to the textural properties of breast meat. Juiciness exhibited a significant quadratic response to the inclusion level, with the highest scores observed at a 3% inclusion. Tenderness also showed a significant quadratic trend, with improvements at both 1.5% and 3% inclusion levels (Table 4).

**TABLE 4:** Sensory evaluations of breast and thigh meat of broiler chicken fed soaked CSK meal.

Meat sensory		Treatments				SEM	*p* value			
		T1	T2	T3	T4		ANOVA	Linear	Quadratic	Cubic
Aroma	Breast	6.38	6.38	6.38	6.34	0.09	0.99	0.81	0.86	0.94
	Thigh	6.38	6.41	6.31	6.34	0.13	0.96	0.74	0.99	0.68

Flavor	Breast	6.38	6.19	6.06	6.38	0.18	0.57	0.88	0.18	0.65
	Thigh	6.38	6.25	6.25	6.13	0.13	0.59	0.17	0.99	0.66

Juiciness	Breast	6.19^ab^	6.75^ab^	6.94^a^	6.13^b^	0.19	0.03	0.98	0.003	0.48
	Thigh	6.38	6.63	6.56	6.31	0.14	0.39	0.71	0.09	0.85

Tenderness	Breast	6.19^a^	6.88^a^	6.81^a^	5.94^b^	0.21	0.02	0.53	0.002	0.95
	Thigh	6.31	6.38	6.69	6.31	0.24	0.64	0.77	0.37	0.39

OA	Breast	6.38	6.56	6.44	6.25	0.15	0.52	0.46	0.21	0.71
	Thigh	6.38	6.56	6.69	6.31	0.14	0.27	0.93	0.06	0.50

*Note:* ANOVA = analysis of variance.

Abbreviation: OA = overall acceptability.

^a,b^Means within a row bearing different superscripts are significantly different.

### 3.3. Blood Biochemistry and Immune Responses

Table 5 presents selected hematological, serum biochemical, and immunological parameters for broilers fed diets incorporating graded levels of soaked CSK meal. The inclusion of soaked CSK meal did not induce any significant (*p* > 0.05) alterations in the WBC count, PCV, MCV, MCH, MCHC, alkaline phosphatase, aspartate aminotransferase, alanine aminotransferase, albumin, urea, or glucose. However, significant differences (*p* < 0.05) were detected for several other parameters. For hematological indices, RBC count and hemoglobin concentration showed significant treatment effects. Orthogonal polynomial contrasts revealed a significant quadratic response for both RBC count and hemoglobin concentration, indicating that these parameters were higher at lower and intermediate dietary levels of soaked CSK meal (T2 and T3) compared to the control treatment (T1) and highest (T4) inclusion levels.

**TABLE 5:** Blood biochemistry and immunological responses of broilers fed different levels of soaked CSK meal.

Parameters	Treatments				SEM	*p* value			
	T1	T2	T3	T4		ANOVA	Linear	Quadratic	Cubic
*Hematology*									
White blood cell × 10^3^/μL	23.19	23.24	24.20	23.18	0.43	0.32	0.66	0.26	0.16
Red blood cell × 10^6^/μL	2.80^b^	3.09^a^	3.13^a^	2.84^b^	0.09	0.04	0.77	0.007	0.83
Hemoglobin, (g/dL)	9.16^b^	10.88^a^	10.83^a^	9.69^ab^	0.37	0.04	0.90	0.004	0.96
Packed cell volume (%)	31.07	34.03	33.84	31.06	0.93	0.06	0.97	0.07	0.90
MCV (fL)	125.46	126.71	126.74	126.21	0.91	0.74	0.57	0.33	0.87
MCH (pg)	34.51	35.20	34.62	33.71	0.54	0.32	0.24	0.15	0.71
MCHC (%)	31.09	31.95	32.00	30.83	0.34	0.07	0.70	0.08	0.79

*Serum Biochemical*									
Alkaline phosphatase (IU/L)	501.24	473.66	496.37	503.22	8.52	0.11	0.53	0.08	0.11
Aspartate aminotransferase (IU/L)	210.25	213.13	240.63	207.13	20.20	0.64	0.84	0.38	0.36
Alanine aminotransferase (IU/L)	18.35	19.38	23.25	18.75	1.60	0.17	0.53	0.13	0.14
Total protein (g/dL)	3.57^b^	3.77^ab^	3.91^a^	3.49^b^	0.07	0.03	0.27	0.04	0.44
Triglycerides (mg/dL)	58.25^a^	47.63^b^	48.55^b^	53.63^ab^	1.95	0.003	0.003	0.09	0.10
Cholesterol (mg/dL)	151.13^a^	131.25^b^	130.25^b^	143.38^ab^	4.70	0.008	0.002	0.20	0.26
Albumin (g/dL)	1.89	1.82	1.98	1.95	0.04	0.11	0.444	0.686	0.225
Total immunoglobulins (mg/dL)	3.41^b^	3.87^a^	3.69^a^	3.26^b^	0.06	0.04	0.62	*p* < 0.001	*p* < 0.001
Globulin (g/dL)	1.68^b^	1.95^a^	1.93^a^	1.54^b^	0.05	0.02	0.05	0.30	0.89
Urea (mg/dL)	13.30	12.84	12.01	10.23	0.79	0.08	0.54	0.42	0.87
Glucose (mg/dL)	202.88	188.13	188.75	189.88	5.57	0.20	0.31	0.37	0.34

*Note:* μl = microliter, dL = deciliter, fL = femtoliters, pg = picograms, U/L = unit per liter, mg = milligram, g = gram.

Abbreviations: MCH = mean corpuscular hemoglobin, MCHC = mean corpuscular hemoglobin concentration, MCV = mean corpuscular volume.

^a,b^Means within a row bearing different superscripts are significantly different.

**FIGURE 1:**
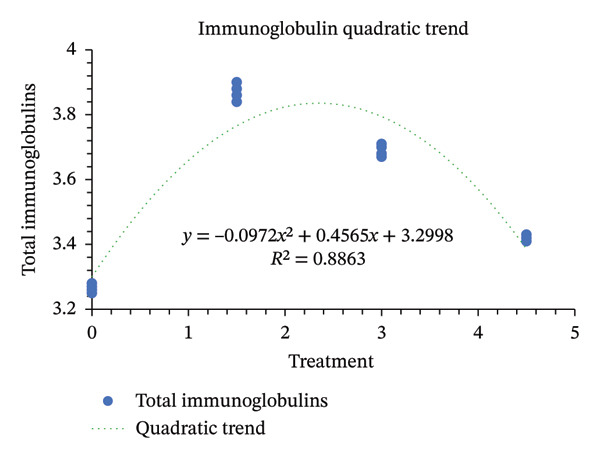
Quadratic effect of the soaked CSK meal level on total immunoglobulins in broilers.

**FIGURE 2:**
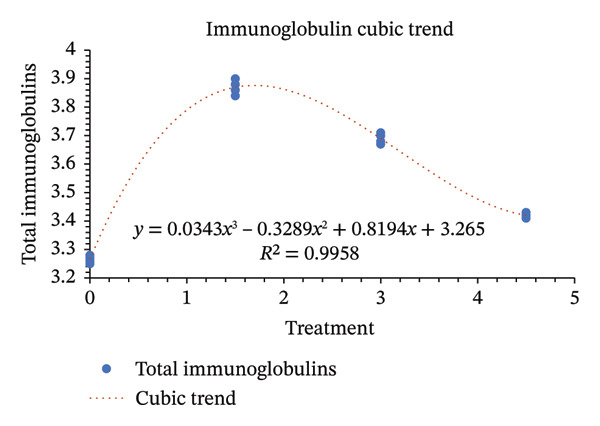
Cubic effect of the soaked CSK meal level on total immunoglobulins in broilers.

Regarding serum biochemical and immunological parameters, significant treatment effects were observed for total protein, triglycerides, cholesterol, total immunoglobulins, and globulin. Total protein showed a significant quadratic response, with the highest level observed in the T3 group. Triglycerides and cholesterol exhibited significant linear decreases with increasing soaked CSK meal inclusion, although the lowest values were recorded in T2 and T3 groups rather than T4. Total immunoglobulins demonstrated significant quadratic and cubic responses, with higher values in T2 and T3 groups compared to those in T1 and T4 (Figure 1 and 2). Globulin levels were also significantly affected by treatment (*p* < 0.05), but polynomial contrasts did not indicate any significant trends.

### 3.4. Cecum Bacterial Load

The cecal microbial analysis of broilers fed varying dietary levels of soaked CSK meal is presented in Table 6. Enumeration of beneficial bacteria indicated that Lactobacilli and Enterococcus spp. populations were unaffected (*p* > 0.05) by dietary treatment, with no significant linear, quadratic, or cubic trends observed. In contrast, the inclusion of soaked CSK meal significantly reduced (*p* < 0.05) E. coli and total coliform counts compared to the control diet. Orthogonal polynomial contrasts revealed a significant quadratic trend for both E. coli and total coliforms, indicating a nonlinear response to increasing CSK meal levels.

**TABLE 6:** Cecum bacterial loads of broilers fed different levels of soaked CSK meal.

Bacterial count	Treatments				SEM	*p* value			
	T1	T2	T3	T4		ANOVA	Linear	Quadratic	Cubic
Enterococcus spp.	7.92	7.96	8.21	8.17	0.09	0.11	0.09	0.68	0.25
Lactobacilli	9.70	10.34	11.05	11.06	0.38	0.08	0.24	0.41	0.66
E. coli	7.86^a^	6.40^b^	5.86^b^	6.68^b^	0.43	0.02	0.46	0.004	0.17
TC	8.62^a^	7.58^b^	6.75^b^	7.63^b^	0.43	0.02	0.28	0.003	0.80

*Note:* Enterococcus spp. = Enterococcus species.

Abbreviations: E. coli = Escherichia coli, TC = total coliform count.

^a,b^Means within a row bearing different superscripts are significantly different.

## 4. Discussion

### 4.1. Physicochemical Quality of Breast and Thigh Muscle

Meat color is a critical quality attribute that profoundly influences consumer acceptance and marketability [50]. The chromatic parameters of lightness (L), redness (a), and yellowness (b) observed in the present investigation for both breast and thigh meat fell within the normative ranges established in the scientific literature [51], indicating that the dietary regimens did not induce any aberrant color development. Notably, significant quadratic and linear modulations in redness (a) were detected in breast and thigh muscle, respectively. Broilers fed lower and a moderate inclusion (1.5%–3.0%) of soaked CSK meal exhibited a significantly higher *a* ^∗^ value than those receiving either the control diet or the highest level of CSK meals. This biphasic response can be attributed to the intricate bioactivity of the phytochemical constituents within CSK, which is quantified to be rich in phenolics and flavonoids [21], along with high levels of glutamic acid [28]. At optimal concentrations, these dietary phenolics and flavonoids are absorbed and incorporated into muscle tissue, where they function as sacrificial antioxidants [28, 52]. By effectively scavenging free radicals and lipid peroxides, they inhibit the oxidative breakdown of myoglobin, thereby improving the cherry‐red pigment of oxymyoglobin and resulting in the significantly higher *a* ^∗^ values recorded for the lower and moderate level of CSK, as reported by Wu et al. [52]. However, the decline in the *a* ^∗^ value at the highest inclusion level (4.5%) within supplemented group seems a shift of the biochemical activity of soaked CSK meal from an antioxidant to word oxidizing entity. This phenomenon is consistent with the principle that polyphenols can exhibit a dualistic role depending on their concentration [53]. Elkady et al. [21] identified a substantial phenolic content of 53.5 mg/g in CSK meal; at excessive concentrations, these compounds may initiate pro‐oxidant reactions rather than antioxidant roles. Such reactions, often involving interactions with metal ions, may foster hydroxyl radical formation. This process can ultimately compromise the phytochemicals’ ability to stabilize the cherry‐red pigment of oxymyoglobin [53].

Dripping loss, a key metric inversely related to water‐holding capacity (WHC), serves as a critical indicator of meat quality. It reflects the meat’s inherent ability to retain water, a property governed by its protein and fat composition. In the present study, the pronounced quadratic reduction in dripping and cooking loss observed at lower and intermediate CSK levels (T2 and T3) for both breast and thigh meat can be attributed to a concomitant increase in protein and a decrease in fat content (Table 3). This finding is consistent with the work of Tilahun et al. [54], who documented a positive correlation between the protein content and WBC, alongside a negative correlation with fat. The proposed mechanism is that a lower fat concentration effectively increases the relative proportion of functional proteins, expanding the matrix of sites available for water immobilization within the muscle structure as reported by Sukmawati et al. [55]. This enhancement of protein content and reduction of fat not only diminishes dripping loss but also directly improves the meat’s overall water‐holding capacity. This effect was further evidenced in the measurement of cooking loss, which also decreased in a significant quadratic manner at lower and intermediate levels of soaked CSK meal. Cooking loss is principally governed by the water content in the meat during the cooking process [54, 56]. The recorded values for breast (27.25%–31.37%) and thigh (29.34%–32.73%) fall within the normative spectrum of 15%–54.5% established for meat products [57]. A lower cooking loss percentage is a desirable quality trait, signifying superior retention of moisture and water‐soluble nutrients, which in turn preserves the product’s nutritional value, juiciness, tenderness, and processing yield.

Similarly, the chemical quality of broiler meat in this study was also assessed through measurements of dry matter content, protein, ether extract, and ash levels. The inclusion of 1.5%–3% soaked CSK meal in the diet significantly altered the macronutrient composition, exhibiting a strong quadratic trend for crude protein in breast meat and for ether extract in both breast and thigh muscles. Specifically, these intermediate soaked CSK levels increased the protein content in breast meat while decreasing the ether extract of both breast and thigh muscle, without significantly affecting dry matter or ash levels. This variation in protein and fat composition may be linked to the seed’s rich phytochemical profile. High‐value amino acids, such as glutamine and all essential amino acids, along with unsaturated fatty acids and flavonoids, may contribute to the higher protein and lower fat deposition in meat, without altering the mineral content or total dry matter. Ibrahim et al. [28] noted that Casimiroa edulis seed protein hydrolysate is particularly rich in glutamic acid, containing 10.25%. Additionally, Reimann et al. [58] found that glutamine promotes the intestinal release of glucagon‐like peptide‐1 (GLP‐1), an enzyme that enhances insulin secretion. This process creates an anabolic environment that favors nutrient utilization for muscle protein synthesis rather than fat deposition.

The other possible reason that may be responsible for the observed improved protein content in breast meat and decreased ether extract in both breast and thigh muscle is aligned with the seed’s rich unsaturated fatty acids. Regarding this issue, Elkady et al. [21] highlighted that gas chromatography analysis of CSK hexane extract revealed high levels of unsaturated fatty acids (94.20%), notably oleic acid (47.00%) and palmitoleic acid (21.00%). These fatty acids positively influence lipid metabolism; palmitoleic acid has been linked to improved lipid profiles and reduced fat accumulation [59]. Furthermore, high‐performance liquid chromatography showed significant flavonoid levels, such as acacetin (2560.78 mg/100 g) and phenolic compounds like pyrogallol (695.98 mg/100 g) in the methanol extract [21]. These bioactive compounds function as antioxidants that may downregulate lipogenic enzymes responsible for fatty acid synthesis while simultaneously upregulating β‐oxidation pathways in the mitochondria, enhancing the breakdown of existing fats for energy [60]. The results of this study agree with those reported by Sukmawati et al. [55], who found significantly higher protein and lower fat deposition in broiler chickens fed fermented jamu‐makarens.

### 4.2. Breast and Thigh Meat Sensory Evaluations

The incorporation of soaked CSK meal in broiler diets appears to enhance breast meat juiciness and tenderness. Notably, both attributes exhibited significant quadratic responses, with improved juiciness observed primarily at a 3% inclusion level, while tenderness was enhanced at both 1.5% and 3% levels, suggesting a favorable effect on consumer acceptance. This finding aligns with the results reported by Gunawan et al. [61], who noted similar sensory enhancements in broiler chickens fed avocado seed flour over a 5‐week period. These benefits may stem from an increased protein concentration in the meat, accompanied by a decrease in ether extracts. According to Al‐Garadi et al. [62], higher protein content in broiler meat correlates with enhanced WHC and lower fat levels. The increase in protein concentration effectively minimizes both dripping and cooking losses by expanding the matrix of sites available for water immobilization within the muscle structure, thereby contributing to the juiciness and tenderness of the meat, as indicated by Sukmawati et al. [55]. Another potential mechanism underlying the observed improvements in juiciness and tenderness may relate to the seed’s abundant polyphenols, particularly phenolic compounds, which play a role in protein regulation. As Ouyang et al. [63] highlighted, these phenolic compounds can coagulate and bind to meat proteins, further inhibiting the release of free water and improving WHC, which sustains the meat’s desirable texture.

### 4.3. Blood Biochemistry and Immunological Responses

Hematological parameters serve as critical indicators of an animal’s physiological status, reflecting its adaptive responses to both internal and external environmental conditions [64]. In the present study, broilers fed diets incorporating graded levels of soaked CSK meal exhibited selected hematological values within established normal ranges. The observed WBC (23.18–24.20 × 10^3^/μL) and RBC (2.80–3.13 × 10^6^/μL) counts were consistent with reference intervals of 12–30 × 10^3^/μL and 2.5–3.5 × 10^6^/μL, respectively [65]. Similarly, the PCV percentages aligned with the normal range (29%–38%) reported by Nworgu et al. [66]. No significant alterations in MCV, MCH, or MCHC were detected across any treatment group, indicating that erythrocyte morphology and hemoglobinization remained unaffected. Notably, dietary inclusion with 1.5% and 3% soaked CSK meal significantly increased the RBC count and hemoglobin concentrations compared to the control (T1) and the 4.5% (T4) groups. This finding is consistent with the work of Al‐Khalaifah et al. [67], who documented a similar hematinic effect in broilers administered graded levels of ginger powder. The overall maintenance of normal hematological values, coupled with enhanced RBC and hemoglobin levels in T2 and T3, suggests that these dietary treatments provided optimal nutrition for hematopoiesis. This interpretation is supported by Oladele et al. [68], who associated suboptimal hematological profiles with nutritional deficiencies. The superior hematological values observed in the T2 and T3 groups indicate improved nutrient utilization and assimilation. This effect may be attributed to the rich profile of bioactive compounds in CSK meal, which includes phenolics, flavonoids, and tannins [21]. These phytochemicals possess potent antioxidant properties that protect erythrocytes from oxidative damage and support their production [69].

The serum biochemical profile and immunological responses provide insight into the metabolic and health status of the birds. The findings of this study indicate that soaked CSK meal supplementation did not induce significant adverse effects on hepatic function markers (AST, ALT, ALP), albumin, or urea. However, it exerted a significant and beneficial modulatory influence on protein metabolism, lipid metabolism, and immune responses. Specifically, total protein and total immunoglobulins were significantly higher in the T2 and T3 groups, indicating an improved protein synthesis and immune response status. Orthogonal polynomial contrasts revealed a significant linear trend for total protein and total immunoglobulins, indicating a consistent increase with an increasing soaked CSK meal inclusion of up to 3%.

The elevated immunoglobulin concentrations in the blood may result from the antioxidant properties CSK meal, which enhanced the birds’ immune response [70]. These findings align with previous research by Corona et al. [24], who reported significantly improved immune response, antioxidant activity, and cell proliferation in the layer birds receiving 5 g/kg of nanoencapsulated Casimiroa edulis fruit seed meal for 1 month. Concurrently, the inclusion of soaked CSK meal at 1.5% and 3% significantly reduced serum cholesterol and triglycerides compared to the control (T1), with values remaining within normal physiological ranges [71]. A significant quadratic trend was observed for both serum cholesterol and triglycerides, suggesting an optimal reduction at the 1.5% and 3% inclusion levels. This hypolipidemic and immunostimulatory effect is likely attributable to the action of bioactive compounds like tannins, present in CSK meal [21], which can form complexes with dietary lipids to reduce their intestinal absorption and concurrently modulate immune function [72]. The observed reductions in serum triglyceride and cholesterol levels align with the findings of Abu Hafsa and Ibrahim [73], who reported that plant‐derived bioactive compounds in dietary grape seed significantly lower triglyceride and cholesterol concentrations. This effect is mechanistically linked to the inhibition of key processes involved in lipid digestion and absorption. Globulin levels were also significantly affected by treatment, but polynomial contrasts did not indicate any significant trends.

### 4.4. Cecum Bacterial Load

Dietary inclusion of soaked CSK meal selectively reduced cecal populations of pathogenic Escherichia coli and total coliforms without affecting beneficial Lactobacilli and Enterococcus spp. This selective antimicrobial effect likely results from interrelated mechanisms following the 24‐h cold‐water soaking pretreatments. Soaking substantially reduces water‐soluble antinutritional factors, including alkaloids, tannins, and oxalates [22, 31]. Lower levels of these compounds preserve intestinal epithelial integrity, attenuate mucosal inflammation, and maintain normal mucus secretion. Consequently, these conditions favor colonization by beneficial commensals such as Lactobacilli while limiting adhesion and proliferation of pathogenic Gram‐negative bacteria such as E. coli [22]. Another possible reason for the significant reduction in cecal E. coli and total coliform counts observed in broilers fed soaked CSK meal may align with the known bioactive properties of Casimiroa edulis seeds. The seeds are a rich source of antimicrobial flavonoids, such as zapotin and 5,6,2′,3′,4′‐pentamethoxyflavone (PMF) [74], as well as various coumarins including imperatorin and heraclenin [27, 74]. These compounds have demonstrated potent biological activities, including vasorelaxation and anticonvulsive effects, often mediated through mechanisms that could indirectly influence microbial growth, such as nitric oxide (NO) release [27, 75]. The nonlinear (quadratic) response of both E. coli and total coliforms to increasing levels of soaked CSK meal suggests that the antimicrobial effect may not follow a linear dose–response pattern; instead, it may plateau or vary with concentration, possibly due to complex interactions among bioactive compounds. It is plausible that these phytochemicals, present in the soaked CSK meal, exert a selective antimicrobial or antibiofilm effect against Gram‐negative pathogens like E. coli, thereby modulating the cecal microbiota in favor of beneficial bacteria without impacting resilient Gram‐positive populations like Lactobacilli and Enterococcus spp., which showed no significant linear, quadratic, or cubic trends. This aligns with findings of Adu et al. [8] who found that the addition of myristica fragrans seed meal significantly reduced pathogenic bacteria without disrupting beneficial microbiota.

## 5. Conclusion

The soaking process rendered CSK meal safe for inclusion in Cobb‐500 broiler diets at levels up to 4.5%, with no observed adverse effects on bird health. However, based on significant quadratic trends across physicochemical, hematological, and gut health parameters, the beneficial and safe inclusion level is refined to 3.0%. Optimal improvements in meat quality, specifically increased breast muscle protein, reduced fat content, enhanced water‐holding capacity, and favorable color attributes, were achieved at inclusion rates of 1.5% and 3.0%. At these levels, soaked CSK meal also improved systemic health, as evidenced by elevated RBCs, hemoglobin, total serum protein, and immunoglobulins, alongside reduced serum cholesterol and triglycerides. Furthermore, it supported gastrointestinal health by selectively decreasing cecal coliform populations without adversely affecting beneficial gut microbiota. Overall, up to 3.0% treated CSK meal can be safely included in broiler diets as a functional ingredient to enhance meat quality and key physiological parameters without adverse effects.

## Author Contributions

Mohammedjuhar Musa Adame was responsible for study conception, research design, formal analysis, data collection and interpretation, initial manuscript preparation, and subsequent revisions. Mengistu Urge Letta contributed to project planning, oversight, guidance, manuscript evaluation, and content refinement. Biazen Abrar Bedru, Meseret Girma Abebe, and Misba Alewi Abdu participated in conceptual development, supervisory functions, critical review, and editorial improvements.

## Funding

This study was partially supported by competitive small‐scale co‐funding grants from Haramaya University (Grant No. HURG_2023_01_01_63) and the Ethiopian Institute of Agricultural Research (Project Code: EIAR‐31‐17‐056).

## Disclosure

All authors have read and approved the final version of the manuscript. The corresponding author (or manuscript guarantor) had full access to all of the data in this study and takes complete responsibility for the integrity of the data and the accuracy of the data analysis.

The corresponding author affirms that this manuscript is an honest, accurate, and transparent account of the study being reported; that no important aspects of the study have been omitted; and that any discrepancies from the study as planned (and, if relevant, registered) have been explained. The funders had no role in the study design, data collection, analysis, or interpretation. They also had no role in the decision to submit the article for publication to a specific journal.

## Ethics Statement

This study received ethical approval (Ref: SARS 052, dated May 24, 2024) from the research and ethics committee of the School of Animal and Range Sciences, Haramaya University. The research procedures strictly adhered to the ethical guidelines outlined in the European Union Directive 2010/63/EU on the protection of animals used for scientific purposes.

## Conflicts of Interest

The authors declare no conflicts of interest.

## Data Availability

The data used in this study are available from the corresponding author upon request to interested parties.

## References

[1] Khan M. T. , Xu C. , Khan S. et al., Dietary Nigella sativa Supplementation Enhances Performance, Carcass Characteristics, Meat Quality, Immune Response, and Gut Health in Broilers, Poultry Science. (2025) 104, no. 10, 10.1016/j.psj.2025.105626. PMC12540292 40774170

[2] Muaz K. , Riaz M. , Akhtar S. , Park S. , and Ismail A. , Antibiotic Residues in Chicken Meat: Global Prevalence, Threats, and Decontamination Strategies: A Review, Journal of Food Protection. (2018) 81, no. 4, 619–627, 10.4315/0362-028X.JFP-17-086. 29537307

[3] Bhosale T. R. , Dhage S. A. , Gaikwad U. S. , Adangale S. B. , and Birari D. R. , Nutritional Perspectives and Innovations in Alternative Feed Resources for Sustainable Poultry Production: A Review, International Journal of Veterinary Sciences and Animal Husbandry. (2025) 10, no. 9, 168–173, 10.22271/veterinary.2025.v10.i9c.2549.

[4] Righi F. , Pitino R. , Manuelian C. L. et al., Plant Feed Additives as Natural Alternatives to the Use of Synthetic Antioxidant Vitamins on Poultry Performances, Health, and Oxidative Status: A Review of the Literature in the Last 20 Years, Antioxidants. (2021) 10, no. 5, 10.3390/antiox10050659. PMC8146777 33922786

[5] Siwach A. , Saini S. , Giri A. , Khatri P. , Kuhad R. C. , and Kumar A. , Sustainable Poultry Feed Formulations From Fruit and Vegetable Residues for Advancing Animal Health, Bioresource Technology Reports. (2025) 30, 10.1016/j.biteb.2025.102159.

[6] Dhama K. , Latheef S. K. , Mani S. et al., Multiple Beneficial Applications and Modes of Action of Herbs in Poultry Health and production—A Review, International Journal of Pharmacology. (2015) 11, no. 3, 152–176, 10.3923/ijp.2015.152.176.

[7] Oloruntola O. D. , Ayodele S. O. , Adeyeye S. A. , Jimoh A. O. , Oloruntola D. A. , and Omoniyi S. I. , Pawpaw Leaf and Seed Meals Composite Mix Dietary Supplementation: Effects on Broiler Chicken’s Performance, *Caecum microflora*, and Blood Analysis, Agroforestry Systems. (2019) 94, no. 2, 555–564, 10.1007/s10457-019-00424-1.

[8] Adu O. A. , Gbore F. A. , Oloruntola O. D. , Aiyegoro O. A. , Ogunbanwo S. T. , and Omoniyi L. A. , The Effects of *Myristica fragrans* Seed Meal and *Syzygium aromaticum* Leaf Meal Dietary Supplementation on Growth Performance and Oxidative Status of Broiler Chicken, Bulletin of the National Research Centre. (2020) 44, no. 1, 10.1186/s42269-020-00396-8.

[9] Getahun A. , Kechero Y. , Yemane N. , Dessie T. , and Esatu W. , Nutritional Evaluation and Potential of Locally Available Alternative Feed Resources for Sustainable Poultry Production: A Case Study of Smallholder Farms in Central and Southern Ethiopia, Tropical Animal Health and Production. (2025) 57, no. 7, 10.1007/s11250-025-04549-7. PMC12279605 40691548

[10] Egbu C. F. , Mulaudzi A. , Motsei L. E. , and Mnisi C. M. , *Moringa oleifera* Products as Nutraceuticals for Sustainable Poultry Production, Agriculture & Food Security. (2024) 13, no. 1, 10.1186/s40066-024-00508-x.

[11] Diaz Carrasco J. M. , Casanova N. A. , and Fernandez Miyakawa M. E. , Microbiota, Gut Health and Chicken Productivity: What is the Connection?, Microorganisms. (2019) 7, no. 10, 10.3390/microorganisms7100374. PMC6843312 31547108

[12] Agustin J. A. , Soto M. , Famiani F. , and Cruz-Castillo J. G. , In Situ Characterization of Fruits and Seeds of a Number of White Sapote (*Casimiroa edulis* Llave and Lex.) Accessions in Mexico, HortScience. (2017) 52, no. 12, 1849–1852, 10.21273/HORTSCI12432-17.

[13] Zeru T. , Characterization and Optimization of Casimiroa (Casimiroa edulis L) Fruit Juice, 2018, 10.20372/nadre:2918.

[14] Satheesh N. , Review on Distribution, Nutritional and Medicinal Values of Casimiroa Edulus Llave-an Underutilized Fruit in Ethiopia, American-Eurasian Journal of Agricultural & Environmental Sciences. (2015) 15, no. 8, 1574–1583.

[15] Assefa F. , Animut G. , Mekasha Y. , and Urge M. , Assessment of the Feeding Potential and Utilization of *Erythrina burana* and *Casimiroa edulis* in Eastern Harerghe Zone of Ethiopia, Livestock Research for Rural Development. (2014) 26, http://www.lrrd.org/lrrd26/5/asse26082.html.

[16] Bekele-Tesemma A. , Useful Trees and Shrubs for Ethiopia: Identification, Propagation and Management for 17 Agroclimatic Zones (Technical Manual No. 6), 2007, RELMA in ICRAF Project, https://www.cifor-icraf.org/knowledge/publication/33785/.

[17] Ahlawat T. R. , Patel N. L. , Patel C. R. , and Agnihotri R. , White Sapote (*Casimiroa edulis* Llave and Lex), Underutilized Fruit Crops: Importance and Cultivation. (2016) Narendra Publishing House, 1353–1367.

[18] Genanew T. Z. , Zegeye A. , and Banjaw B. T. , Characterization and Optimization of *Casimiroa edulis* Fruit Juice Using Response Surface Methodology (RSM), International Journal of Agricultural Science and Food Technology. (2022) 8, no. 2, 101–107, 10.17352/2455-815X.000150.

[19] Adame M. M. , Letta M. U. , Bedru B. A. , Terfa S. G. , Abebe M. G. , and Abdu M. A. , Effects of Dietary Inclusion of Casimiroa (*Casimiroa edulis* L) Seed Kernel Meal on Growth Performances, Carcass Yield and Profitability of Broilers Chicken, 2025, 10.21203/rs.3.rs-6498700/v1.

[20] Tun K. , Aminah N. , Kristanti A. , Aung H. , and Takaya Y. , Natural Products Isolated From Casimiroa, Open Chemistry. (2020) 18, no. 1, 778–797, 10.1515/chem-2020-0128.

[21] Elkady W. M. , Ibrahim E. A. , Gonaid M. H. , and El Baz F. K. , Chemical Profile and Biological Activity of *Casimiroa edulis* Non-Edible Fruit’s Parts, Advanced Pharmaceutical Bulletin. (2017) 7, no. 4, 655–660, 10.15171/apb.2017.079. 29399557 PMC5788222

[22] Jatutu S. S. , Bello K. M. , and Kalla D. J. U. , Effect of Differently Processed Mango Seed Kernel Meal on Performance of Broiler Chickens, Nigerian Journal of Animal Production. (2024) 1214–1220.

[23] Awaad A. S. , Maitland D. J. , and Moneir S. , New Alkaloids From *Casimiroa edulis* Fruits and Their Pharmacological Activity, Chemistry of Natural Compounds. (2007) 43, no. 5, 576–580, 10.1007/s10600-007-0196-9.

[24] Corona L. R. D. , Rodriguez M. E. M. , Perez L. M. A. , Yerena A. R. , Martinez Preciado A. H. , and Reyes-Becerril M. , Immunostimulant Effects of Diet Supplementation with Yellow (*Pouteria campechiana*), White (*Casimiroa edulis*), and Black (*Diospyros digyna*) Sapote Nanocapsules on Laying Hens: In Vitro and In Vivo Study, Tropical Animal Health and Production. (2023) 55, no. 6, 10.1007/s11250-023-03778-y. 37851183

[25] Magos G. A. and Vidrio H. , Pharmacology of Casimiroa Edulis; Part I. Blood Pressure and Heart Rate Effects in the Anesthetized Rat, Planta Medica. (1991) 57, no. 1, 20–24, 10.1055/s-2006-960008. 2062952

[26] Bertin R. , Garcia-Argaez A. , Martinez-Vazquez M. , and Froldi G. , Age-Dependent Vasorelaxation of *Casimiroa edulis* and *Casimiroa pubescens* Extracts in Rat Caudal Artery in Vitro, Journal of Ethnopharmacology. (2011) 137, no. 1, 934–936, 10.1016/j.jep.2011.06.027. 21726621

[27] Froldi G. , Bertin R. , Secchi E. , Zagotto G. , Martinez-Vazquez M. , and Garcia-Argaez A. , Vasorelaxation by Extracts of *Casimiroa* spp. in Rat Resistance Vessels and Pharmacological Study of Cellular Mechanisms, Journal of Ethnopharmacology. (2011) 134, no. 3, 637–643, 10.1016/j.jep.2011.01.008. 21236328

[28] Ibrahim N. , Hawary S. E. , Mohammed M. , Ali S. , Kandil Z. , and Refaat E. , Chemical Compositions and Hypoglycemic Activities of the Protein and Mucilage of *Casimiroa edulis* (Llave and Lex) Seeds and Fruits, Journal of Applied Pharmaceutical Science. (2019) 9, no. 10, 10.7324/JAPS.2019.91011.

[29] Lozoya X. , Romero G. , Olmedo M. , and Bondani A. , Farmacodinamia de Los Extractos Alcohólico y Acuoso de la Semilla de Casimiroa Edulis, Archivos de Investigacion Medica. (1977) 8, no. 2, 145–154, https://pubmed.ncbi.nlm.nih.gov/334096/. 334096

[30] Kebede M. , Sharma J. , Tana T. , and Nigatu L. , Effect of Plant Spacing and Weeding Frequency on Weed Infestation, Yield Components, and Yield of Common Bean (*Phaseolus vulgaris* L.) in Eastern Ethiopia, East African Journal of Sciences. (2015) 9, no. 1, 1–14, 10.20372/eajs.v9i1.254.

[31] George O. S. , Kingsley C. , and Ekine O. A. , Effects of Dietary Inclusion of Avocado Seed Meal (*Persea americana*) on the Carcass Yield and Haematological Profile of Broiler Chickens, Nigerian Journal of Animal Production. (2020) 47, no. 2, 82–88, 10.51791/njap.v47i2.102.

[32] Aoac International , Official Methods of Analysis of AOAC International, 2005, 18th edition.

[33] Wiseman J. , Feeding of Non-ruminant Livestock, 2013, Elsevier.

[34] Nrc (National Research Council) , Nutrient Requirements of Poultry, 1994, 9th edition, National Academies Press, 10.17226/2114.

[35] Tareen M. H. , Wagan R. , Siyal F. A. et al., Effect of Various Levels of Date Palm Kernel on Growth Performance of Broilers, Veterinary World. (2017) 10, no. 2, 227–232, 10.14202/vetworld.2017.227-232. 28344407 PMC5352849

[36] American Meat Science Association , Meat Color Measurement Guidelines, Champaign, IL, 2016.

[37] Torres Filho R. de A. , Cazedey H. P. , Fontes P. R. , Ramos A. de L. S. , and Ramos E. M. , Drip Loss Assessment by Different Analytical Methods and Their Relationships With Pork Quality Classification, Journal of Food Quality. (2017) 2017, no. 1, 1–8, 10.1155/2017/9170768.

[38] Hoffman L. C. , Muller M. , Cloete S. W. P. , and Schmidt D. , Comparison of Six Crossbred Lamb Types: Sensory, Physical and Nutritional Meat Quality Characteristics, Meat Science. (2003) 65, no. 4, 1265–1274, 10.1016/S0309-1740(03)00034-2. 22063769

[39] Damaziak K. , Stelmasiak A. , Riedel J. et al., Sensory Evaluation of Poultry Meat: A Comparative Survey of Results from Normal Sighted and Blind People, PLoS One. (2019) 14, no. 2, 10.1371/journal.pone.0210722. PMC6353138 30699202

[40] Bernard F. F. , Joseph G. Z. , and Jain N. C. , Schalm’s Veterinary Hematology, 2000, 5th edition, Wiley.

[41] Irizarry-Rovira A. R. , Cowell R. L. , Avian and Reptilian Clinical Pathology (Avian Hematology and Biochemical Analysis), Veterinary Clinical Pathology Secrets, 2004, Elsevier, St. Louis, 282–313.

[42] Young D. S. and Friedman R. B. , Effects of Disease on Clinical Laboratory Tests, 2001, 1, 4th edition, AACC Press.

[43] Douglas J. , Weiss K. , and Wardrop K. J. , Veterinary Hematology, 2010, 6th edition, Blackwell Publishing Ltd.

[44] Mondesire R. R. , Thrall M. A. , Baker D. C. , Campbell T. W. et al., Immunodiagnostics and Other Emerging Diagnostic Techniques, Veterinary Hematology and Clinical Chemistry, 2004, Lippincott Williams and Wilkins, 55–68.

[45] Petričević V. , Dosković V. , Lukić M. et al., Effect of Peppermint (*Mentha piperita* L.) in Broiler Chicken Diet on Production Parameters, Slaughter Characteristics, and Gut Microbial Composition, Large Animal Review. (2021) 27, 103–107, https://r.istocar.bg.ac.rs/handle/123456789/736.

[46] Cengiz Ö. , Koksal B. H. , Yagin O. et al., Influence of Dietary Prebiotic Addition on Digestibility and Intestinal Microflora of Young Male Broiler Chickens Exposed to Delayed Feed Access After Hatch, International Journal of Poultry Science. (2012) 11, no. 6, 408–416, 10.3923/ijps.2012.408.416.

[47] Abolfathi M. E. , Tabeidian S. A. , Foroozandeh Shahraki A. D. , Tabatabaei S. N. , and Habibian M. , Comparative Effects of n-Hexane and Methanol Extracts of Elecampane (*Inula helenium* L.) Rhizome on Growth Performance, Carcass Traits, Feed Digestibility, Intestinal Antioxidant Status and Ileal Microbiota in Broiler Chickens, Archives of Animal Nutrition. (2019) 73, no. 2, 88–110, 10.1080/1745039X.2019.1581027. 30821191

[48] Willis W. L. , King K. , Isikhuemhen O. S. , and Ibrahim S. A. , Administration of Mushroom Extract to Broiler Chickens for Bifidobacteria Enhancement and Salmonella Reduction, Journal of Applied Poultry Research. (2009) 18, no. 4, 658–664, 10.3382/japr.2008-00101.

[49] Oyeagu C. E. , Mlambo V. , Muchenje V. , and Marume U. , Effect of Dietary Supplementation of Aspergillus Xylanase on Broiler Chicken’s Performance, Iranian Journal of Applied Animal Science. (2019) 9, no. 4, 693–708, https://journals.iau.ir/article_669381.html.

[50] Adeyemi K. D. , Shittu R. M. , Sabow A. B. et al., Comparison of Myofibrillar Protein Degradation, Antioxidant Profile, Fatty Acids, Metmyoglobin Reducing Activity, Physicochemical Properties and Sensory Attributes of Gluteus Medius and Infraspinatus Muscles in Goats, Journal Of Animal Science And Technology. (2016) 58, 1–17, 10.1186/s40781-016-0124-2. 27307997 PMC4908769

[51] Zhang L. and Barbut S. , Effects of Regular and Modified Starches on Cooked Pale, Soft, Exudative; Normal; and Dark, Firm, Dry Chicken Breast Meat Batters, Poultry Science. (2005) 84, no. 5, 789–796, 10.1093/ps/84.5.789. 15913192

[52] Wu H. , Bak K. H. , Goran G. V. , and Tatiyaborworntham N. , Inhibitory Mechanisms of Polyphenols on Heme Protein-Mediated Lipid Oxidation in Muscle Food: New Insights and Advances, Critical Reviews in Food Science and Nutrition. (2022) 64, no. 15, 4921–4939, 10.1080/10408398.2022.2146654. 36448306

[53] Serra A. , Foggi G. , Buccioni A. et al., Dietary Supplementation with Natural Antioxidants: Assessment of Growth Performance and Meat Quality in Broiler Chickens, Poultry Science. (2024) 103, no. 3, 10.1016/j.psj.2023.103421. PMC10831253 38244263

[54] Tilahun M. , Urge M. , Girma M. , Ameha N. , Gadissa S. , and Welay K. , Effects of Dietary Inclusion of Black Cumin Seed (*Nigella sativa* L.) Meal on Meat Quality, Hematology, and Serum Biochemical and Antioxidant Status of Broiler Chickens, Scientific African. (2026) 32, 10.1016/j.sciaf.2026.e03430.

[55] Sukmawati N. M. S. , Ardika I. N. , and Melati N. P. Y. , Blood Lipid Profile and Meat Quality of Broiler Chickens Fed Fermented Jamu Makarens, International Journal of Fauna and Biological Studies. (2024) 11, no. 1, 16–22, 10.22271/23940522.2024.v11.i1a.1002.

[56] Warner R. D. , The Eating Quality of Meat–IV Water-Holding Capacity and Juiciness, Lawrie’s Meat Science, 2017, 8th edition, Elsevier, 419–457, 10.1016/B978-0-08-100694-8.00014-5.

[57] Soeparno , Meat Science and Technology, 2015, 6th edition, Gadjah Mada University Press.

[58] Reimann F. , Williams L. , da Silva X. G. , Rutter G. A. , and Gribble F. M. , Glutamine Potently Stimulates Glucagon-Like Peptide-1 Secretion From Glutag Cells, Diabetologia. (2004) 47, no. 9, 1592–1601, 10.1007/s00125-004-1498-0. 15365617

[59] Ding X. , Giannenas I. , Skoufos I. , Wang J. , and Zhu W. , The Effects of Plant Extracts on Lipid Metabolism of Chickens—A Review, Animal Bioscience. (2023) 36, no. 5, 679–691, 10.5713/ab.22.0272. 36397703 PMC10164473

[60] Beylot M. , Effects of Inulin-Type Fructans on Lipid Metabolism in Man and in Animal Models, British Journal of Nutrition. (2005) 93, no. 1, 163–168, 10.1079/bjn20041339. 15877890

[61] Gunawan A. , Erwan E. , Rodiallah M. , and Zumarni Z. , The Physical Quality of Broiler Chicken Meat Given Basal Ration Containing Avocado Seed Flour (*Persea americana* Mill), Jurnal Ternak. (2020) 11, no. 2, 78–85, 10.30736/jt.v11i2.86.

[62] Al-Garadi M. A. , Alhotan R. A. , Hussein E. O. et al., Phytogenic and Prebiotic Feed Additives as Antibiotic Substitutes: Effects on Broiler Meat Quality, Organ Properties, and Production Efficiency when Fed Standard and Reduced Energy and Protein Diets, Cogent Food & Agriculture. (2025) 11, no. 1, 10.1080/23311932.2025.2531964.

[63] Ouyang K. , Xu M. , Jiang Y. , and Wang W. , Effects of Alfalfa Flavonoids on Broiler Performance, Meat Quality, and Gene Expression, Canadian Journal of Animal Science. (2016) 96, no. 3, 332–341, 10.1139/cjas-2015-0132.

[64] Chowdhury S. R. , Smith T. K. , Boermans H. J. , and Woodward B. , Effects of Feed-Borne Fusarium Mycotoxins on Hematology and Immunology of Laying Hens, Poultry Science. (2005) 84, no. 12, 1841–1850, 10.1093/ps/84.12.1841. 16479939

[65] Bounous D. and Stedman N. , Feldman B. F. , Zinkl J. G. , and Jain N. C. , Normal Avian Hematology: Chicken and Turkey, Schalm’s Veterinary Hematology, 2000, Wiley, 1147–1154.

[66] Nworgu F. C. , Ogungbenro S. A. , and Solesi K. S. , Performance and Some Blood Chemistry Indices of Broiler Chicken Served Fluted Pumpkin (*Telferia occidentalis*) Leaves Extract Supplement, Journal of American-Eurasian Agricultural and Environmental Science. (2007) 2, no. 1, 90–98.

[67] Al-Khalaifah H. , Al-Nasser A. , Al-Surrayai T. et al., Effect of Ginger Powder on Production Performance, Antioxidant Status, Hematological Parameters, Digestibility, and Plasma Cholesterol Content in Broiler Chickens, Animals. (2022) 12, no. 7, 10.3390/ani12070901. PMC8996832 35405889

[68] Oladele S. B. , Ayo J. O. , Esievo K. A. N. , and Ogundipe S. O. , Seasonal and Sex Variations in Packed Cell Volume, Haemoglobin Total Protein of Indigenous Duck in Zaria Nigeria, Journal of Tropical Bioscience. (2001) 1, no. 1, 84–88.

[69] Kumar M. , Saurabh V. , Tomar M. et al., Mango (*Mangifera indica* L.) Leaves: Nutritional Composition, Phytochemical Profile, and Health-Promoting Bioactivities, Antioxidants. (2021) 10, no. 2, 10.3390/antiox10020299. PMC7920260 33669341

[70] Abd El-Hack M. E. , El-Saadony M. T. , Elbestawy A. R. et al., Hot Red Pepper Powder as a Safe Alternative to Antibiotics in Organic Poultry Feed: An Updated Review, Poultry Science. (2022) 101, no. 4, 10.1016/j.psj.2021.101684. PMC8850793 35168162

[71] Saleh A. A. and Ebeid T. A. , Feeding Sodium Selenite and Nano-Selenium Stimulates Growth and Oxidation Resistance in Broilers, South African Journal of Animal Science. (2019) 49, no. 1, 176–183, 10.4314/sajas.v49i1.20.

[72] Erwan E. , Afriadi A. , Rodiallah M. , Irfan I. , and Ibrah W. , Effects of Supplementation of Saviotan Feed (Chestnut Tannin) on Blood Parameters and Yolk Cholesterol Concentration in Japanese Quails (*Coturnix japonica*), Journal of World’s Poultry Research. (2023) 13, no. 3, 317–322, 10.36380/jwpr.2023.34.

[73] Abu H. S. H. and Ibrahim S. A. , Effect of Dietary polyphenol-rich Grape Seed on Growth Performance, Antioxidant Capacity and Ileal Microflora in Broiler Chicks, Journal of Animal Physiology and Animal Nutrition. (2018) 102, no. 1, 268–275, 10.1111/jpn.12688. 28295656

[74] Bertin R. , Chen Z. , Martínez-Vázquez M. , García-Argaéz A. , and Froldi G. , Vasodilation and Radical-Scavenging Activity of Imperatorin and Selected Coumarinic and Flavonoid Compounds From Genus Casimiroa, Phytomedicine. (2014) 21, no. 5, 586–594, 10.1016/j.phymed.2013.10.030. 24309287

[75] Baisch A. L. , Urban H. , and Ruiz A. N. , Endothelium-Dependent Vasorelaxing Activity of Aqueous Extracts of Lyophilized Seeds of *Casimiroa edulis* (Aece) on Rat Mesenteric Arterial Bed, Journal of Ethnopharmacology. (2004) 95, no. 2-3, 163–167, 10.1016/j.jep.2004.06.018. 15507330

